# A Study on the Geometric and Kinematic Descriptors of Trajectories in the Classification of Ship Types

**DOI:** 10.3390/s22155588

**Published:** 2022-07-26

**Authors:** Yashar Tavakoli, Lourdes Peña-Castillo, Amilcar Soares

**Affiliations:** Department of Computer Science, Memorial University of Newfoundland, St. John’s, NL A1B 3X5, Canada; lourdes@mun.ca (L.P.-C.); asoaresjunio@mun.ca (A.S.)

**Keywords:** trajectory, descriptor, classification, ship, feature engineering, feature selection, model interpretation, knowledge discovery

## Abstract

The classification of ships based on their trajectory descriptors is a common practice that is helpful in various contexts, such as maritime security and traffic management. For the most part, the descriptors are either geometric, which capture the shape of a ship’s trajectory, or kinematic, which capture the motion properties of a ship’s movement. Understanding the implications of the type of descriptor that is used in classification is important for feature engineering and model interpretation. However, this matter has not yet been deeply studied. This article contributes to feature engineering within this field by introducing proper similarity measures between the descriptors and defining sound benchmark classifiers, based on which we compared the predictive performance of geometric and kinematic descriptors. The performance profiles of geometric and kinematic descriptors, along with several standard tools in interpretable machine learning, helped us to provide an account of how different ships differ in movement. Our results indicated that the predictive performance of geometric and kinematic descriptors varied greatly, depending on the classification problem at hand. We also showed that the movement of certain ship classes solely differed geometrically while some other classes differed kinematically and that this difference could be formulated in simple terms. On the other hand, the movement characteristics of some other ship classes could not be delineated along these lines and were more complicated to express. Finally, this study verified the conjecture that the geometric–kinematic taxonomy could be further developed as a tool for more accessible feature selection.

## 1. Introduction

As several marine tracking technologies have become more prevalent in reporting the positions of ships, movement mining practices, in particular the classification of ship trajectories, have emerged as an active research area. By the classification of ship trajectories, we mean the supervised learning problem of assigning the correct ship type to a trajectory. Ship trajectory classification is useful for identifying illegal activities, imposing regulations, managing navigation, maintaining biodiversity, extracting routes, and detecting anomalies.

Movement is usually expressed using trajectories. A raw trajectory consists of either an ordered sequence of spatial pairs, each signifying a 2D position (latitude and longitude), or an ordered sequence of spatiotemporal triplets, each signifying a 2D position along with a time stamp. Trajectories in higher dimensions are also conceivable. According to [1], it is possible to work with raw trajectories directly or to instead employ certain random variables called descriptors, which are defined as random variables for either spatial or spatiotemporal sequences. Therefore, in essence, a descriptor is a scalar value that measures a certain aspect of the trajectory (e.g., the average speed or straightness) [2,3,4] and can serve as a feature or attribute in the classification terminology. There are a number of benefits to using descriptors, for example, higher performance (precision, speed, scalabilty, etc.) compared to using methods that only operate with raw trajectories [3,5,6,7,8,9,10,11,12]. Furthermore, descriptors are intuitive to work with and produce explainable data mining artifacts [3,5,9,10]. Data mining with descriptors also demands less memory usage compared to raw trajectories [3] and hence, descriptors allow for techniques that are impractical for raw trajectories [9]. Finally, descriptors are more suitable for visualization [5]. A *general* descriptor can be computed just from the trajectory, regardless of the presence or absence of any contextual information, such as geographical information regarding the location of the trajectory. Since general descriptors were the focus of this study, we usually drop the word “general” from the term throughout this paper.

There have been several studies that have applied descriptors to the classification of ships based on their trajectory. In particular, Sanchez Pedroche et al. [13] focused on the binary problem of distinguishing between fishing and non-fishing ships using a support vector machine. The authors cited food safety and biodiversity issues surrounding illegal fishing as the reasons for solely focusing on fishing ships in their classification problem. Regarding their target descriptors, the study employed the descriptive statistics of the speed, distance, and course of the vessels in question. Likewise, Sheng et al. [14] used a support vector machine to tackle the binary problem of distinguishing between fishing and cargo ships. The authors of that article chose this specific problem merely to showcase their method while indicating the significant differences between the trajectories of fishing ships and those of cargo ships. As well as descriptors relating to speed and distance, and course, that article benefited from an array of descriptors that captured the turn features of the vessels. The authors of [15] proposed a feature fusion network (among several others) to achieve accurate multiclassification, with an emphasis on handling imbalanced data. Eight types of ships were investigated in that article, including cargo ships, passenger ships, oil tankers, towing ships, container ships, pilot ships, law enforcement ships, and fishing ships. The list of descriptors that was used in this study extended beyond those of the two previous studies to also include curvature and orientation angle. Relying on the same descriptors, the authors of [16] compared the performances of different ensemble learners for the binary classification between fishing boats and pleasure crafts as an example of ship classification using an imbalanced dataset. The main descriptive statistic that they used for the descriptors was the change rate. The authors of [17] employed recurrent neural networks to classify different types of fishing boats, in which the authors employed an array of descriptors that measured the jaggedness of the trajectories, as well as the more common descriptors relating to speed and distance. Finally, Kraus et al. [18] tackled the classification of ship types problem using random forests. The ship types that were considered for classification in that study were fishing ships, passenger ships, cargo ships, and tankers. In terms of descriptors, the authors employed the ratio of the distance between the start and end of the trajectory to the total distance traveled, which reflected how much the shape of the trajectory deviated from a straight line. Furthermore, the article benefited from more crafty descriptors, such as the main course ratio, which measured the zigzagging behavior of the trajectory, and speed ratios, which reflected the ratios of the total length of the trajectory segments in which the ship was moving slowly or quickly to the length of the entire trajectory.

From a taxonomical perspective, all of the above research articles employed *geometric* and/or *kinematic* descriptors. Geometric descriptors operate on spatial sequences, whereas kinematic descriptors operate on spatiotemporal sequences. In the literature, these two types of descriptors are always grouped separately in one way or another, albeit under different names. For example, in [9], the descriptors that were recognized as kinematic and temporal (both of which fit the definitions that we put forward for kinematic descriptors) were grouped differently from the descriptors that were recognized as shape (which was equivalent to what we call geometric descriptors). However, [19] recognized this latter group as geometric shape [20] but, on the other hand, recognized several classes of descriptors, some of which fell under the definition of geometric and the rest under the definition of kinematic descriptors. The authors of [21] used the term geometric with the same connotations as the present article; however, they employed the term motorial for the descriptors that we refer to as kinematic.

The first research question (RQ) that we addressed in this study (**RQ1**) was *how do the predictive performances of geometric and kinematic descriptors compare for the classification of ship types*? The answer to this question could be valuable to inform the selection of descriptors or to assess the importance of acquiring the spatiotemporal components of a trajectory. This could be particularly important as the temporal components of a trajectory could be missing or unreliable. For example, in the case of a trajectory that was inferred from an aerial image containing the traces of a moving object [22,23], the temporal component would be missing altogether or in the cases of a trajectory that was based on a sequence of images with a low temporal resolution (for example, refer to [24,25]), the kinematic descriptors may not be reliable. Additionally, it is common for the time reporting mechanism of a GPS device to glitch and cause “uncertainty” [2]. Nevertheless, spatial components may not be reliable either, as the geometries of trajectories that belong to the same moving object can vary, depending on the reporting device [26]. In addition, trajectories that are based on surveillance cameras can involve obscure spatial properties (for example, refer to [27]). As another example, the position reporting mechanism of a GPS device could be faulty and again cause uncertainty [2]. Some research articles exist in the literature that are related to **RQ1**. The authors of [16,21,28] raised the fact that in their respective classification contexts, one type of descriptor, either geometric or kinematic, was generally favored for the classification of ships [16] or land vehicles [21,28]. More importantly, however, none of these articles produced any rationales as to why one type was favored over the other. The authors of [29] provided evidence for the claim that some geometric descriptors produce a more accurate classification of land vehicles; however, this study included a limited number of descriptors. The shortcomings of the literature are three-fold: the lack of quantitative comparisons between a wide array of geometric and kinematic descriptors, which could also include trajectory descriptors that are still unused within the context of the classification of ship types, for the most common ship types. This study tackled all of these shortcomings.

Our second research question (**RQ2**) was *can geometric and kinematic descriptors characterize the differences between how various types of ships move?* In this regard, a number of research articles on ship classification have provided the importance factors of several descriptors with respect to the underlying models [7,12,30]. However, theses articles have not provided a comprehensible interpretation of exactly what the important descriptors signify in terms of movement characterization. The range of employed descriptors has also been limited. In contrast, this study ascribed the movement differences between certain ship types to geometric and kinematic descriptors and provided a simple explanation for those movement differences.

It can be speculated that the cohesive and distinct definitions of geometric and kinematic descriptors could prove useful for feature selection in ship classification problems. A prior knowledge of predictors in general could guide practitioners to pick relevant predictors from groups of similar variables to reduce redundancy [31,32,33,34,35,36]. Accordingly, our last research question (**RQ3**) was *does the geometric–kinematic taxonomy induce groups of similar descriptors?* The authors of [19,29,37] resorted to using similarity between descriptors for feature selection. However, none of these studies focused on the ship classification problem. Furthermore, the similarity analyses in the mentioned studies were not along the geometric–kinematic line. In this article, on the other hand, we discovered groups of similar geometric and kinematic descriptors for all of the common ship classification problems that are currently under study and paved the way for more refined discoveries.

In summary, the contributions of our study with respect to our research questions are the following.

1. The examination of a wide range of ship classification problems using comprehensive sets of geometric and kinematic descriptors and a quantitative determination of the problems for which geometric or kinematic descriptors can deliver acceptable results on their own and those that rely on both sets of descriptors;
2. The uncovering of the potential of geometric and kinematic descriptors as stand-alone attributes in movement characterization, along with the identification and analysis of the complex movements of certain ship classes for which neither set is enough for movement characterization;
3. The verification of the conjecture that similar descriptors in ship classification emerge in line with a geometric–kinematic taxonomy and the development of a comprehensive set of descriptors.

The rest of the article is structured as follows. First, in Section 2, we present an overview of the data and descriptors that were used in this study. In the same section, we also lay out the details of the steps that we took and the technical choices we selected to produce our answers for each of the three research questions (RQs) that we posed. Section 3 presents the actual answers to the research questions. Finally, in Section 4, we present a summary of our findings and their implications within their respective contexts. Section 4 also includes remarks about the components of the study for which further extensions or improvements would be conceivable.

## 2. Materials and Methods

The datasets and classification problems are described in Section 2.1 and Section 2.2, respectively. The list of kinematic and geometric descriptors are detailed in Section 2.3. Finally, we dedicate Section 2.4, Section 2.5 and Section 2.6 to describing how this study tackled **RQ1**, **RQ2**, and **RQ3**, respectively.

### 2.1. Data

**Figure 1:**
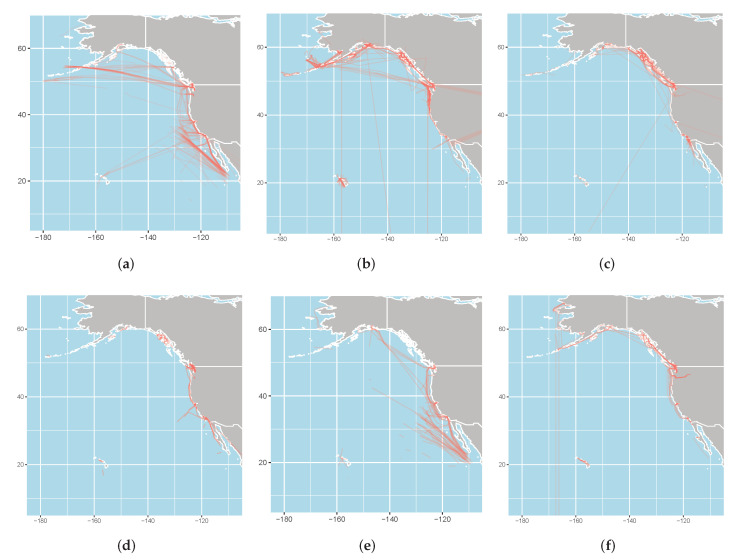
The trajectories that were contained in the raw data according to class, as portrayed on their respective regions (note the invalid/glitchy trajectories that were later removed): (**a**) cargo ships; (**b**) fishing ships; (**c**) passenger ships; (**d**) sailing ships; (**e**) tankers; (**f**) towing ships.

The source of the trajectories that were used in this study was the Automatic Identification System (AIS), which is currently the most commonly used position reporting system in the marine industry. We focused on the common classes of ships: cargo ships, tankers, towing ships, fishing ships, passenger ships, and sailing ships (along with their subclasses). Before discussing the data, it should be noted that ship classes usually consist of subclasses. For example, container ships can vary drastically in size, resulting in a wide variety of movement characteristics that all bundled into a single class. So, when choosing our data, we focused on the entire Pacific region of Mexico, United States, and Canada (which is delineated by a latitude between −180 and −110 and a longitude between 10 and 70) over the summer season during the 5-day pre-COVID window of 26 June 2019 to 30 June 2019. We relied on expert knowledge when choosing the area, season, and dates to ensure that the data reflected all of the subclasses of cargo ships, tankers, towing ships, fishing ships, passenger ships, and sailing ships. We used the Maritime Mobile Service Identity (MMSI), which is a unique number for every ship, in each AIS message (which are available for free at https://marinecadastre.gov/ais/ (accessed by the authors on 13 June 2021)) to identify the ship trajectories. After filtering out the data with invalid MMSI values (for example, those with non-numeric characters), the AIS messages with the same MMSI values formed a single trajectory. Figure 1 depicts the trajectories for each class, as delineated by the MMSI values. It is clear from the figure that the data contained some invalid/glitchy trajectories, such as in-land and/or unusually long and straight trajectories. We also removed all such instances from the data. Table 1 breaks down the data according to class. It also shows that the data were moderately balanced.

### 2.2. Classification Problems

To make a comprehensive result possible, we looked at all of the existing ∑i=266i=57 classification problems for the six classes. There was one caveat though: the treatment of all 57 problems would be impractical if approached crudely. Thus, we relied on certain measures, which are described below, to ensure that the complexity would not overwhelm the study.

### 2.3. Descriptors

The descriptors that were used in this study were an aggregate of the descriptors that are commonly used in the context of ship classification, along with some cutting-edge descriptors from other fields.

Table 2 lists the geometric descriptors that were used in this study. They reflected several geometric properties of trajectories, from those as simple as length to more complex properties, such as sinuosity. Notably, we borrowed multiple geometric descriptors from the aviation domain (e.g., those regarding the convex hull of a trajectory) [9,10], which are yet to be used in the marine domain. Therefore, as a by-product, this study also revealed the potential of these descriptors for use in the context of ship classification.

For the kinematic descriptors, we relied on the trajectory segmentation that was proposed by [14], in which a ship trajectory is partitioned into *anchored off*, *turning*, and *straight sailing* segments. Table 3 lists the kinematic descriptors that were used in the present study.

### 2.4. Investigation into the Predictive Performances of Geometric and Kinematic Descriptors (RQ1)

For each classification problem within the spectrum of 57 problems, we needed three models for our performance analysis. These included models that were solely based on geometric descriptors and those that were solely based on kinematic descriptors, as well as benchmark models that were based on the aggregate of both geometric and kinematic descriptors. There were some feasibility concerns:

1. Since our approach involved hundreds of models, tuning could become a bottleneck;
2. The performance assessment of so many models was time-consuming;
3. The modeling method had to guarantee that the models that were based on both geometric and kinematic predictors (in this paper, we refer to descriptors as predictors in the context of modeling), as the benchmark, did not underperform the models that were solely based on either geometric or kinematic descriptors as not all the modeling methods meet this requirement: some modeling methods perform worse in the presence of multicolinearity between additional predictors and some perform worse when additional predictors contain irrelevant predictors.

A modeling method that overcomes all of the above-mentioned concerns is random forest. With regard to the first concern, random forest represented an “outstanding” out-of-the-box method [40,41] that could help to streamline the modeling phase of this study and save us from the cumbersome tweaking of the hyperparameters of so many models. In most cases, out-of-the-box random forest models perform comparatively well and further optimization only slightly boosts their performance [40,41]. In this study, our model was generated with default hyperparameters: 500 trees and $\sqrt{p}$ predictors sampled at each split, where *p* is the total number of predictors. Another advantage of random forest is its facilitation of an effective and efficient performance estimation. As our data were balanced and thus, accuracy was a suitable performance metric, we used test/prediction error (accuracy). Practitioners usually estimate prediction error using cross-validation, which is carried out separately from the modeling; however, a by-product of random forest modeling is the so-called out of bag error (OOB), which is a very good approximation of the prediction error [41]. Hence, random forest saved us from the overhead of cross-validation, which would have been impractical for so many models. Furthermore, random forest models that were based on both geometric and kinematic predictors could serve as benchmarks due to the robustness of random forest in the presence of both irrelevant variables [41] and multicolinearity between the variables [42]. Finally, applying a single modeling method across the board provided an even ground for the many comparisons that were involved in this study.

### 2.5. Investigation into the Capabilities of Geometric and Kinematic Descriptors for Movement Characterization (RQ2)

The second research question (**RQ2**) was formulated to determine whether geometric and kinematic descriptors could characterize the differences between the movements of different ship classes. **RQ2** could be phrased as an estimation of the interpretive performances of geometric and kinematic descriptors. This study approached this question on two levels: interpretation based on the predictive performances of the descriptors and interpretation based on interpretable models. We now explain what each level entailed.

As touched upon in Section 1, the group of geometric descriptors and the group of kinematic descriptors enjoyed distinct and intuitive interpretations. As such, the performances of the geometric and kinematic descriptors could shed light on the differences between the movement characteristics of different ship classes. For example, for the classification problem of distinguishing between ship class A and ship class B, when the predictive performance of the geometric descriptors approaches the benchmark, then the geometric descriptors can discriminate between class A and class B well. This inference would characterize the differences between ship class A and ship class B as *shape*-related. Likewise, when the predictive performance of the kinematic descriptors approaches the benchmark, then the differences between ship class A and ship class B would be *motion*-related. Based on this argument, we looked at the predictive performances of the geometric or kinematic descriptors to examine the classification problems for which they approached the benchmark. In particular, we took a deeper look at binary problems as differences between characteristics are more comprehensible when only comparing two classes.

At the second level, we refined the characterization of classification problems with geometric or kinematic characteristics. This was possible by interpreting the models of the corresponding geometric and kinematic classification problems. A model that was based on all of the available descriptors might not be interpretable due to the existence of redundancy. The concept of *optimal feature sets* let us produce a model that was based on a reduced set of predictors, which manifested both low redundancy and an acceptable predictive power. According to [43], an optimal set of features exclusively consists of either strongly relevant variables and/or weakly relevant but non-redundant variables. This assertion, although correct, needed more restrictions here since our study relied on *highly* interpretable models and we were not able to accommodate any groups of highly correlated (hence, strongly redundant) variables. So, in this study, we limited the optimal set of features to either strongly relevant but not strongly redundant variables and/or weakly relevant but non-redundant variables.

For the question of the interpretive performances of the geometric and kinematic predictors, we built a model using the optimal feature set for each classification problem with geometric or kinematic characteristics, the interpretation of which provided a refined characterization of the differences between the movement of ships in the different classes that were involved in our study. Since finding the optimal feature set was tedious, we limited our quest to the classification problems that showed the highest geometric or kinematic characteristics. In hybrid problems, on the other hand, for which pure well-performing geometric or kinematic models were not available, we tried to gauge the hybrid characteristics of the problem. More precisely, we explored how much the geometric and kinematic predictors *relied* on each another to achieve the classification results. To do this, we likewise relied on the interpretation of interpretable models for select classification problems in which both geometric and kinematic predictors performed worse on their own.

The process outlined above also entailed certain other details, which we now explain. Based on their definitions, relevancy and non-redundancy (or the dissimilarity to other variables) rendered each variable a member of the optimal feature set of a problem. Regarding the order in which we investigated relevancy and the similarity of variables, the similarity analysis took precedence over the relevancy analysis for measuring the relevancy of a smaller set of variables (after removing similar variables), which was not only much more practical but also much more reliable due to the minimal correlation. As a first step, we defined what constituted similarity among the descriptors. Researchers and practitioners usually employ correlations (particularly Pearson’s correlation coefficient) to measure the similarity between two variables. In this study, correlations were the basis for our similarity analysis (the Section 4 touches on some other techniques as well); however, Pearson’s correlation coefficient was not an appropriate choice in this study. Before presenting the reasons, we first remind the reader that we sought generalization in all areas of this study. Accordingly, we used confidence intervals to make inferences in our similarity analysis, which Pearson’s correlation coefficient would not allow since our sample was not normal. Although ,according to the central limit theorem, valid Pearson correlation coefficients can be derived for sufficiently large non-normal samples [44,45], the corresponding confidence intervals would still be unreliable. This is rooted in the instability of the Fisher’s $z^{\prime }$ transformation (which underlies the Pearson’s correlation coefficient calculation of confidence intervals) with respect to non-normal data [46]. Therefore, since confidence intervals are integral to the inferential component of this study, we dismissed Pearson’s correlation coefficient. According to [46], only the Spearman rank-order correlation coefficient and the rank-based inverse normal transformation produce reliable confidence intervals for non-normal data. Here, we opted for the more popular Spearman rank-order correlation coefficient. Rank-based correlation measures account for both linear and non-linear correlations. By choosing the Spearman over Pearson’s correlation coefficient, we traded a more accurate identification of linear correlations for a more reliable inference and the detection of some non-linear correlations. The trade-off particularly stood out when we noted that a certain degree of monotone correlation between some of the descriptors (for example, the area and perimeter of the convex hull) was trivial, whereas the linear correlations were obscured and worthy of further research. Finally, the Spearman rank-order correlation coefficient can be employed in different ways to measure similarity; here, we opted for a simple yet operational definition in which the absolute value of the Spearman rank-order correlation coefficient between two variables represented their similarity. Therefore, a greater absolute value meant that the variables were more similar to each other.

With the definition of similarity in place, we now clarify how we thoroughly investigated the similarity (or more accurately, the similarity strength) between a multitude of variables in this study. This was carried out using clustering, which detects groups of similar variables. Practitioners and researchers usually use hierarchical clustering for the clustering of distance/similarity matrices. We also employed hierarchical clustering for the link heights that are exclusive to this method (which represent the distances between clusters), which also helped us with our similarity analysis. Additionally, it should be mentioned that we did not detect any tangible differences between the agglomerative (which is run on different linkage criteria) and divisive hierarchical clustering methods. Since the number and make-up of the clusters ultimately determine the optimum feature set, the optimum feature set ties in with optimum clustering. Therefore, we were able to measure the quality of any given clustering. The two main parameters that, when maximized, guarantee the quality of a given clustering are the compactness of the clusters and the distances between them. Since our similarity analysis relied on both compactness and separation, we opted for the popular silhouette method, which optimizes both parameters simultaneously [47]. Based on this definition, we could then take a representative feature from each cluster and call the collection an optimal feature set. Before doing so, however, it should be noted that clustering is blind to response variables. As a result, the size of the optimum feature set that is suggested by the clustering result may not lead to a model that produces a reasonable performance. This circumstance would violate the relevancy factor and the definition of an optimal feature set. To ensure that our optimal feature set merited the name, we built a model using a set of representatives and ensured that the model was appropriate, in that the performance was not significantly compromised in comparison to a model with no excluded descriptors. This model was also used to study the underlying feature spaces of the problems under study. When choosing the representatives, we followed a few guidelines:

- To ensure that the representatives well encapsulated the characteristics of the cluster and not the other clusters, the representatives needed to manifest strong similarity bonds to the rest of the host cluster and weak similarity bonds to the predictors in foreign clusters as much as possible;
- Weak models have weak ties to the ground truth; so, representatives that led to even higher performance models produced more reliable interpretations and thus, took precedence over the others;
- A predictor could take precedence over the others when its definition was more intuitive and hence, interpretable;
- Where applicable, both geometric and kinematic predictors were well represented in the selected set of predictors.

We could then perform our model interpretation using the optimal feature sets at our disposal. As the first step, we gauged the quality of the optimal feature sets by examining the strength of the similarity bonds within the clusters (compactness) and between the clusters (separation), mainly using descriptive statistics and visualization techniques. We also extended our analysis to the whole population using statistical inference. Note that each cluster signified an underlying feature, which in itself might or might not have a straightforward expression. The next phase was model interpretation. The first useful tool that we employed here was feature importance. Regarding the calculation method for importance factors, we used the preferred model-agnostic permutation approach for model-specific measures. The reason behind this preference was the superior stability of the results that were produced by the permutation approach [48]. However, we could deepen our insights into the models under study by using techniques that are commonly known as *global interpretation techniques* among practitioners and researchers, which shed light on how predictors produce their results. For binary problems, we resorted to *2D partial dependence plots* (PDPs) [49,50], which provide readily understandable results for two classes that depict how the probability of positive/negative predictions changes for the first class according to the values that are taken by the pair of variables. For the second class, the PDP is just an inversion of the first PDP. The number of PDPs that need to be included is decided by the number of important variables. For multiclass problems, the use of PDPs is impractical; thus, we used *Kruskal–Wallis H test* for important predictors in order to understand the entanglement of the geometric and kinematic descriptors when producing the classification results. The H test, in principle, signifies to what extent the effect of an independent variable on the value of a dependent variable changes when other independent variables change as well. The results of the H test are between 0 and 1: the closer the value is to 0, the straighter the effect of the dependent variable and vice versa. For example, consider loosing weight as the dependent variable with the two independent variables of working out and diet. We know that both independent variables help in losing weight, but no diet considerably diminishes the results of working out, whereas diet alone has more of a *straight* effect in that it is more effective in the absence of working out than the other way around. Therefore, working out would have a higher H test result in comparison to diet (the H test is not commutative). The H test provides a clear picture of interaction levels (or lack thereof) between several variables within a model.

### 2.6. Investigation into the Group Similarity Induced by Geometric and Kinematic Descriptors (RQ3)

Our last research question (**RQ3**) entailed the conjecture that *universally* similar descriptors emerge as either geometric or kinematic types. By universally similar, we mean that they are similar for *every* classification problem. It should be noted that with the addition/removal of data points (which was induced by the inclusion/exclusion of classes in this study), the correlation (or similarity) between a pair of random variables can change. As such, similar descriptors for a certain classification problem may not necessarily be similar for another classification problem. To verify whether universally similar groups of geometric or kinematic descriptors exist, we used a *data-driven* approach. Data-riven approaches are more reliable since our problem involved complex and hidden relationships between the dependent and independent variables [51].

Therefore, we investigated whether it was valid to assert that geometric and kinematic descriptors that are similar exist, regardless of the inclusion/exclusion of classes. To do so, we first needed to explore which groups of descriptors could generally be similar and second, we needed to verify this conjecture. To form the conjecture, we looked at the similarity between the descriptors for each class in isolation and pinpointed descriptors that emerged as similar. We employed 2D multidimensional scaling (MDS) [34,52], which provides a visual scheme and hence, better suited the purposes of this study. The MDS basically arranged the descriptors onto a cartography-like map according to the corresponding similarity matrices. A follow-up inspection of the distances between the descriptors on the map easily led to the verification of the conjecture. The conjecture was then validated by verifying the similarity for all of the possible problems.

## 3. Results

In accordance with the approach that was outlined in the previous section, we answer the three research questions that were posed earlier in this section.

### 3.1. The Predictive Performance of Geometric and Kinematic Descriptors (RQ1)

**Figure 2:**
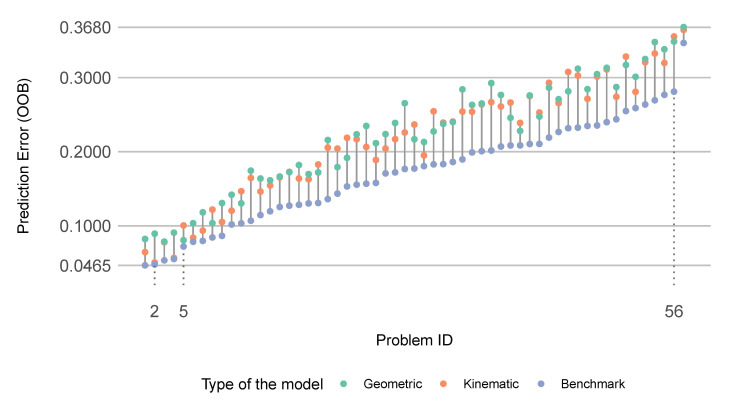
The performance profiles of the random forest models that were based on the 3 different sets of predictors for each of the 57 classification problems. The problems were sorted based on the OOB errors of the models that were based on both geometric and kinematic predictors.

**Figure 3:**
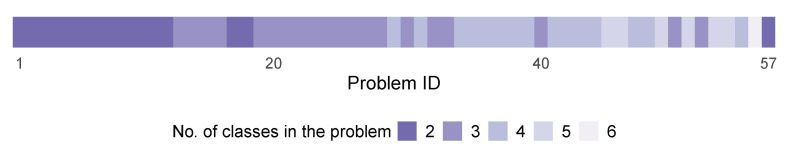
The numbers of classes that were present in the problems, which were labeled and sorted according to increasing levels of hardness. The classification problems that involved more classes tended to be harder, with some exceptions.

To evaluate the predictive performance of the geometric and kinematic descriptors, we built three random forest models for each of the 57 problems: (i) one that was based on geometric predictors; (ii) one that was based on kinematic predictors; and (iii) one that was based on a combination of both geometric and kinematic descriptors (benchmark). As depicted in Figure 2, the benchmark model helped to us assemble a more expressive visualization by ordering and labeling the problems according to the ascending OOB error (prediction error) of the models that were based on the combination of geometric and kinematic predictors. Therefore, the problems that are on the horizontal axis in Figure 2 were ordered in terms of the corresponding *hardness* (i.e., the lower the performance of the benchmark model, the harder the corresponding classification problem). As the number of classes that were involved in the problems grew, so did the hardness. However, as portrayed in Figure 3, there were exceptions. For example, the hardest problem (represented by Problem ID 57) was the binary classification between cargo ships and tankers, while the second hardest problem (Problem ID 56) was the classification problem that consisted of all six classes. The easiest problem was the binary classification between sailing ships and cargo ships.

The best model was the benchmark model for sailing ships vs. cargo ships, with an OOB error of 0.0465 and the worst model was the geometric descriptor-based model for cargo ships vs. tankers, with an OOB error of 0.368. The benchmark models, for the most part, had lower OOB errors than the geometric/kinematic descriptor-based models, as portrayed in the first and second rows of Table 4. On average, the benchmark models outperformed the geometric-descriptor based models by more than 5% and the kinematic descriptor-based models by more than 4.5%. The best performance enhancement that was achieved by the benchmark models was almost 9.5%. This particular problem is highlighted as Problem ID 56 in Figure 2. This enhancement made sense because Problem ID 56 comprised all of the classes, which entailed a variety of movement nuances.

The third row of Table 4 suggests that the benchmark models, on average, outperformed the best alternative model (based on either geometric or kinematic descriptors) by more than 5.5%.

By excluding the benchmark models, we could compare the predictive performances of the geometric and kinematic descriptor-based models, as portrayed in the fourth row of Table 4. On average, the kinematic descriptor-based models outperformed the geometric descriptor-based models by 0.6%; however, the performance gap could reach up to 4%. A comparison of the means and medians also showed that the distribution of OOB errors was negatively skewed, meaning that more kinematic descriptor-based models outperformed geometric descriptor-based models than the other way around.

### 3.2. The Interpretive Performance of Geometric and Kinematic Descriptors (RQ2)

The predictive performance of the geometric and kinematic descriptors demonstrated that, in some cases, it was be possible to ascribe the differences between the characteristics of the ship classes to either the shape of the trajectories or the motion of the moving ships. For example, as indicated by the first and second rows of Table 4, the geometric and kinematic descriptor-based models could approach the performance of the benchmark models with a difference of 0.8% (highlighted in Figure 2 as Problem ID 5) and 0.1% (highlighted in Figure 2 as Problem ID 2), respectively. So, the differences between cargo ships and passenger ships could be characterized as geometric (Problem ID 5) and the difference between sailing ships and tankers could be characterized as kinematic (Problem ID 2). In some problems (such as Problem ID 56, which consisted of all six classes), this characterization did not hold.

**Figure 4:**
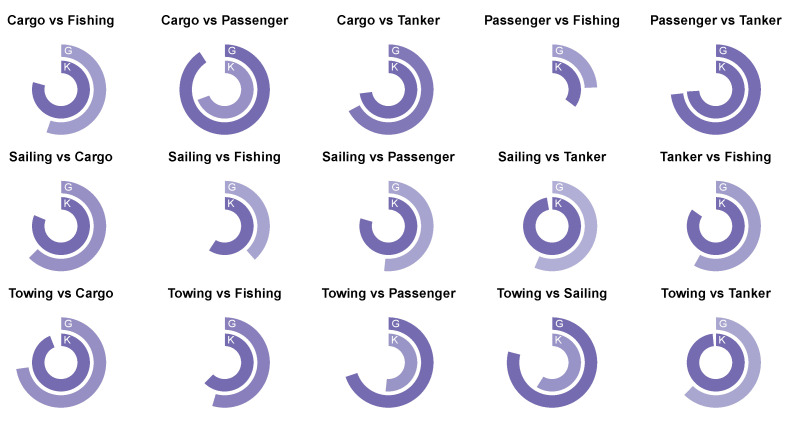
The relative performances of the geometric and kinematic descriptor-based models with reference to the corresponding benchmark models for binary problems. The outer ring depicts the performance of the geometric descriptor-based models and the inner ring depicts the kinematic descriptor-based models.

As we suggested earlier, the characterization of movement differences in binary problems was more intelligible. Therefore, we now present and decipher the predictive performance of the descriptors with respect to the characterization of movement differences in these problems. To compare how effectively the geometric and kinematic predictors could characterize the movement differences in different binary problems, we calculated the relative predictive performances by dividing the performance (OOB error) of the geometric/kinematic descriptor-based models by that of the corresponding benchmark models. For example, when the predictive performance of a geometric descriptor-based model was 0.8 and the performance of the corresponding benchmark model was 0.9, then the relative predictive performance of the geometric descriptor-based model was 89% with reference to the benchmark model. This metric was between 0.92432 and 0.9984334 for all of the geometric and kinematic descriptor-based models. Since the first significant digits did not provide any information, we discarded them to produce Figure 4.

Accordingly, we could argue that the movement of cargo ships differed from that of passenger ships much more geometrically than kinematically. Likewise, the movement of sailing ships differed from tankers much more kinematically than geometrically. In cases with hybrid characteristics, some possessed more of a geometric nature than kinematic (cargo ships vs. passenger ships, towing ships vs. passenger ships, and towing ships vs. sailing ships) and vise versa (cargo ships vs. fishing ships, sailing ships vs. passenger ships, sailing ships vs. tankers, tankers vs. fishing ships, and towing ships vs. tankers). It would be fair to assert that cargo ships and tankers, as well as passenger ships and tankers, showed both geometric and kinematic discriminative characteristics, whereas passenger ships and fishing ships did not manifest either geometric or kinematic discriminative characteristics.

In light of Section 2.5, we now refine the geometric and kinematic movement characterization of some sample classification problems. We selected a case with no prevalent geometric or kinematic characteristics to interpret how the geometric and kinematic descriptors collaborated while discriminating between ship classes. We selected three problems and chose the two best geometric and kinematic descriptor-based models for each of those problems. Figure 2 depicts the kinematic descriptor-based model for Problem ID 2 (sailing ships vs. tankers), which had the lowest difference from the corresponding benchmark model in terms of OOB error. Similarly, Figure 2 depicts the geometric descriptor-based model for Problem ID 5 (cargo ships vs. passenger ships), which had the lowest difference from the corresponding benchmark model in terms of OOB error. Finally, Figure 2 depicts Problem ID 56, for which the benchmark model significantly outperformed the best alternative geometric or kinematic descriptor-based models and in which all six classes were involved.

**Figure 5:**
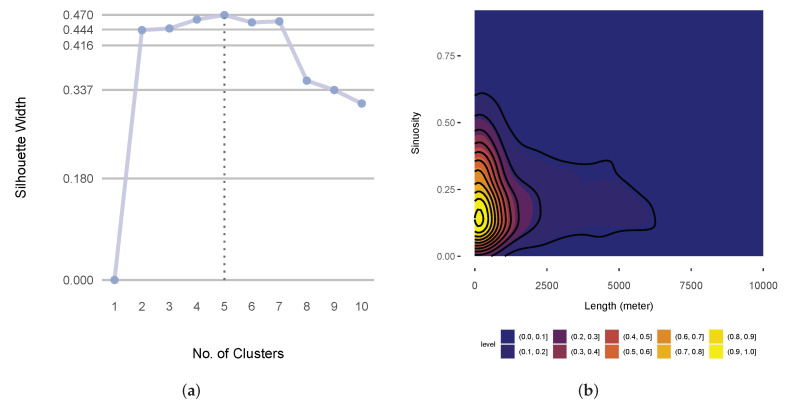
(**a**) The silhouette scores for the different numbers of clusters from the similarity matrix that consisted of the geometric descriptors; (**b**) a kernel density plot of the sinuosity and length of the similarity matrix that consisted of the geometric descriptors.

**Figure 6:**
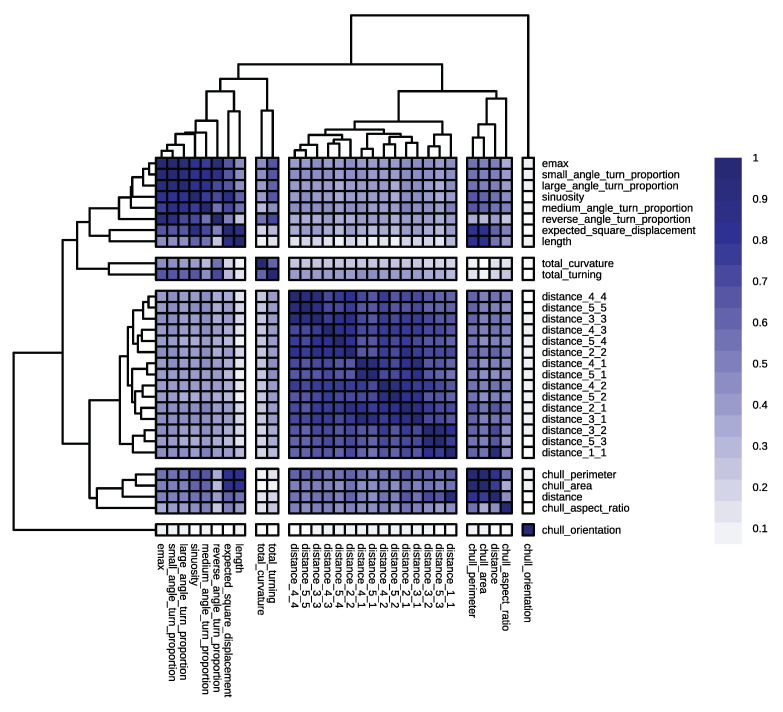
The hierarchical clustering of the similarity matrix that consisted of the geometric descriptors. The similarity index for a pair of descriptors was between 0 and 1.

Starting with the classification of cargo ships and passenger ships and in accordance with the outlined methodology, we first performed hierarchical clustering on the similarity matrix that consisted of the geometric descriptors. The silhouette method suggested five as the optimum number of clusters (Figure 5a). Figure 6 illustrates the five clusters that were produced by the hierarchical clustering of the selected similarity matrix.

The model that was based on the set of cluster representatives that consisted of length, convex_hull_aspect_ratio, total_turning, total_curvature, geometry_1_1, and convex_hull_orientation produced an OOB error rate of 9.85% compared to the 8.33% OOB error rate that was produced by the model that was based on the geometric predictors (as shown in Figure 2). This slight performance decline, along with the fact that the descriptors were very mildly correlated, implied that the representative predictors could constitute an optimal feature set and that they decently represented the feature space of the problem, which we now demonstrate. In the first cluster (top left corner), we observed that the turn proportions monotonically increased and decreased in direct or reverse correlation with each other, respectively. The length had the weakest bond within the cluster; however, from an inferential point of view, the descriptor that showed large confidence ranges (near 0.1) across the board was length. So, in the worst-case scenario in which the similarity was off by 0.05 (when the correlation coefficients were in the middle of the confidence interval with an accuracy of at least three significant digits), the similarity bond could fall well below 50%.

The second cluster solely consisted of total_turning and total_curvature. These two descriptors did not necessarily represent the same attributes in all problems; for example, even though the total curvature signified how much the vessel maintained its course, a vessel could make several twists and turns while maintaining its course. When these two descriptors were in the same cluster, this could imply that cargo and passenger ships mostly turned to correct their course. The correlation between total_turning and total_curvature was around 0.61 and the half width of the confidence interval was around 0.03, which meant that the similarity bond between these two descriptors was at least 58% within the population.

The distance geometries that formed the third cluster also emerged as monotonically similar. In fact, their relationships were strictly direct, so they all increased and decreased together. Table 5 demonstrates that the strength of the similarity was fairly significant across the board, reaching a maximum of 94%. This was only partially intuitive: the finer and coarser signatures of the distance geometries were directly correlated since when the whole population is tortuous, so are the parts (and vice versa). However, it was interesting that similarity existed between the signatures at the same level. This implied that, for example, when the first half of a trajectory that belonged to a cargo ship or a passenger ship was somewhat straight, so was the second half (note that none of the correlation coefficients were negative). The maximum half width of the confidence intervals for the distance geometries was 0.03 since the correlation in that cluster did not fall below 0.57. In the worst-case scenario, the similarity bond stayed at least as strong as 54% within the population.

In the fourth cluster, some descriptors were present that were expected a priori. This was due to the obvious monotone relationship between distance, convex_hull_perimeter, and convex_hull_area. The existence of this relationship was further demonstrated by the corresponding negligible widths of the confidence intervals. However, convex_hull_aspect_ratio was a rather weak link, not only within the population but also within the sample. The correlation between distance and convex_hull_aspect_ratio was −0.37, which weakly implied that the longer the trajectories of cargo and passenger ships within the sample, the shorter and narrower their turns. The convex_hull_orientation descriptor was solely present in the fifth cluster. It was not apparent how important a feature it was to the classification problem at hand.

Finally, it should be mentioned that there was a rather substantial similarity between some of the descriptors in the first cluster and those in the fourth cluster. This was reasonable because, for example, the more sinuous the trajectory, the larger the area of the convex hull that was covered. However, convex_hull_aspect_ratio and distance were better choices for more interpretable models as they were less akin to the descriptors in the first cluster.

**Figure 7:**
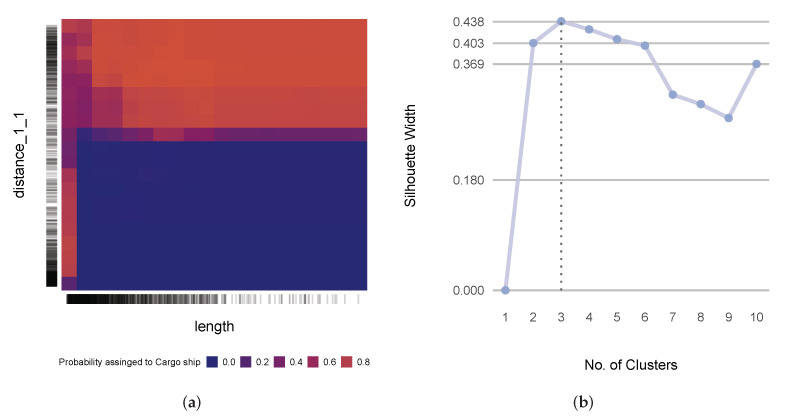
(**a**) The PDP of distance_1_1 and length for geometric descriptor-based models; (**b**) the silhouette scores from the different numbers of clusters for the kinematic descriptor-based models.

With this clearer picture of the underlying features of the problems, we could try to find expressions for them. All of the clusters, except for the first one, seemed to be sufficiently self-expressive. To comprehend the underlying features better, we could roughly deduce that two attributes measured the same underlying feature by taking sinuosity as the representative of *turn* descriptors within the cluster and length as the representative of *magnitude* descriptors. From a representation point of view, the representatives monotonically (although not necessarily linearly) increased and decreased in relation to each other. Regarding the types of ships in question, this relation was inverse, as indicated by their correlation coefficient of −0.42. The nature of the relationship between these two descriptors for cargo ships and passenger ships is depicted by the kernel density plot in Figure 5b. As outlined earlier, we next explore the models using their importance factors and PDPs. The permutation importance factors for distance_1_1, length, chull_aspect_ratio, chull_orientation, and total_curvature were 0.37, 0.14, 0.6, 0.4, and 0.4, respectively. This showed how integral distance_1_1 and length were. The PDP in Figure 7a shows how these two most important predictors interacted. The PDP implied that the more tortuous the trajectory, the lower the probability that it belonged to a cargo ship (except when the trajectory was very short and there was also a high density of data points, as indicated by the sidebar, which perhaps reflected near-shore activities). The pronounced and highly contrasted marginal probabilities (ranging from almost 0 to almost 1) further demonstrated the efficacy of the these two predictors in distinguishing between cargo ships and passenger ships.

**Figure 8:**
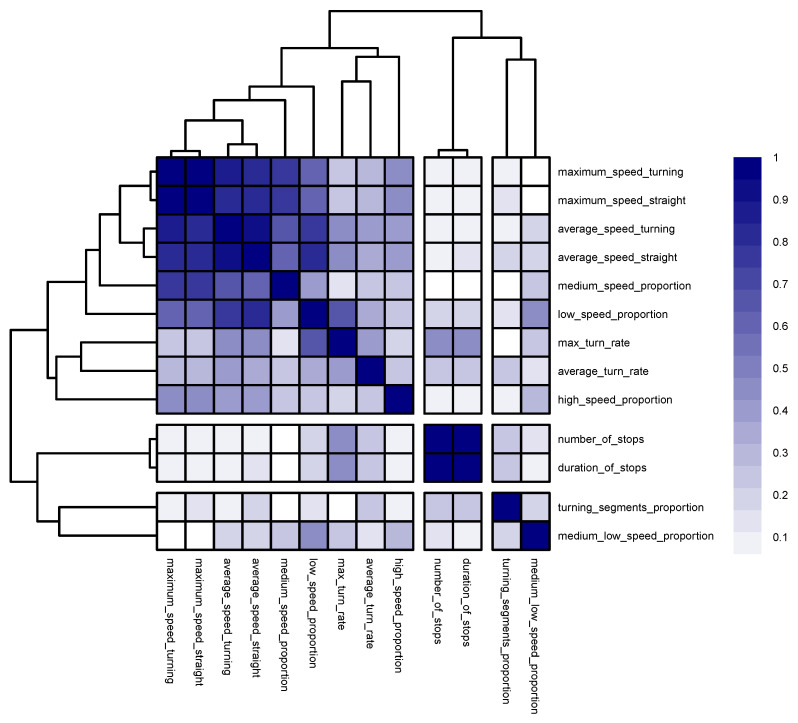
The hierarchical clustering of the similarity matrix for the kinematic descriptor-based models. The similarity index for a pair of descriptors was between 0 and 1.

Now, we move on to the kinematic problem that we picked to investigate, which involved the classification between sailing ships and tankers. Figure 8 portrays the corresponding similarity matrix with three clusters. The silhouette method suggested that the optimal number of clusters was three, as illustrated in Figure 7b.

The model that was based on maximum_speed_straight, number_of_stops, and medium_low_speed_proportion as the cluster representatives produced an OOB error rate of 6.22% in comparison to the 5.05% OBB error rate that was produced by the model that was based on the full set of kinematic descriptors. The rather insubstantial drop in performance, along with the fact that the selected predictors were non-redundant, attested to the reasonable quality of the defined optimal feature set.

When looking into the first cluster, a subcluster emerged very strongly. The subcluster consisted of maximum_speed_straight, maximum_speed_turning, average_speed_straight, and average_speed_turning. The average similarity factor within the cluster was 0.87, with a standard deviation of 0.6. The maximum half width of the confidence interval was 0.02, which implied that the similarity bond among these descriptors was strong within the population as well (in fact, the relationship between these descriptors was directly colinear). The max_turn_rate, average_turn_rate, and high_speed_proportion descriptors had looser ties, with an average similarity factor of 0.32 and a standard deviation of 0.1. The half width average was 0.06.

The second cluster, consisting of duration_of_stops and number_of_stops, was quite strong within both the sample and the population, with a similarity bond of 0.97 and a maximum half width of 0.005.

Unlike the second cluster, the third cluster, consisting of turning_segments_proportion and medium_low_speed_proportion, was quite weak within the sample and the population, with a similarity bond of 0.21 and a half width of 0.07.

**Figure 9:**
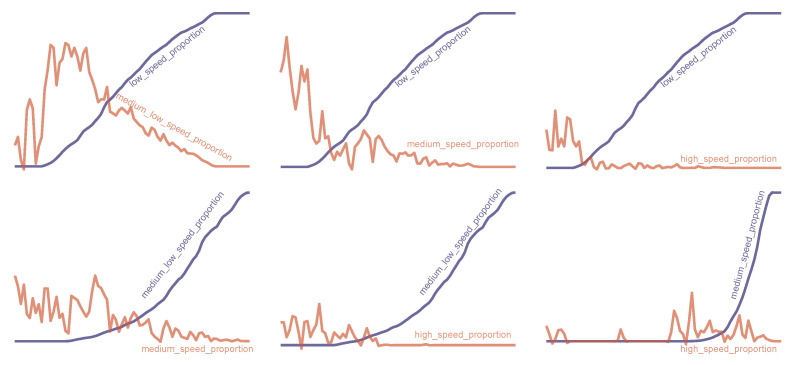
A portrayal (with a 5% smoothing) of the weak monotone relationships between the pairs of speed proportion descriptors in the kinematic descriptor-based model.

One rather interesting observation regarding the kinematic descriptors for this problem was the weak similarity bond among the descriptors that represented the speed proportions as the geometric proportions in the previous problem manifested much stronger similarity bonds with each another. We investigated the absence of strong monotone relationships within the pairs of speed proportion descriptors by monotonically ordering one descriptor and plotting a second descriptor against the first, as portrayed in Figure 9. We clearly observed that only the first two descriptors manifested monotone similarity.

**Figure 10:**
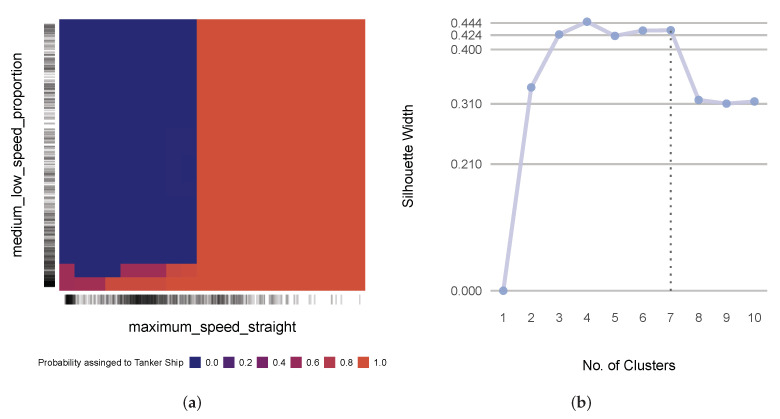
(**a**) The PDP of maximum_speed_straight and medium_low_speed_proportion for the kinematic descriptor-based model; (**b**) the silhouette scores from the different numbers of clusters for the hybrid problem.

The permutation importance factors for maximum_speed_straight, medium_low_ speed_proportion, and number_of_stops were 0.2, 0.17, and 0.04, respectively. The PDP in Figure 10a depicts how the prediction was performed using the two most important predictors in the model. We observed that maximum_speed_straight split the probability space, in which a maximum speed that was higher than a certain threshold meant that the trajectory in question almost certainly belonged to a tanker. Trajectories with maximum speeds of less than the threshold belonged to sailing ships, for the most part. Here, medium_low_speed_proportion played a corrective role for trajectories with a maximum speed that was less than the threshold, which could still be tankers when the medium_low_speed_proportion was small enough. The side bar shows that a significant number of trajectories belonged to this region.

**Figure 11:**
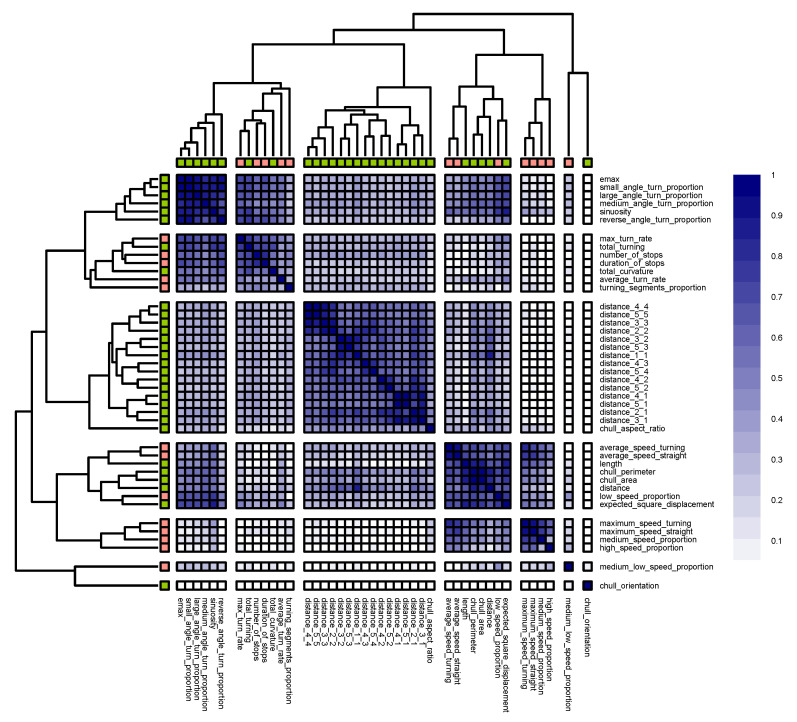
The hierarchical clustering of the similarity matrix for the hybrid problem: 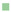 geometric predictors; 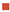 kinematic predictors. The similarity index for a pair of descriptors was between 0 and 1.

As we planned, we now examine the problem that consisted of all six classes with the full set of descriptors, which lent it a hybrid characteristic. Figure 11 illustrates the similarity matrix of the descriptors, which were clustered into seven groups. Note that the optimum number of clusters was four, as shown in Figure 10b. However, four clusters did not result in a model that had an acceptable compromise in terms of performance. Therefore, we opted for seven clusters as the second best choice, which achieved an acceptable performance in comparison to that of the benchmark model.

The model that was based on sinuosity, number_of_stops, distance_1_1, chull_ perimeter, maximum_speed_straight, medium_low_speed_proportion, and chull_ orientation as cluster representatives produced an OOB error rate of 31.37%. This showed less than a 4% jump in error rate compared to the 27.49% error rate that was produced by the benchmark model. By assuming that the error jump was tolerable with so many predictors not included, we could also assume the relevancy of the selected representatives of the underlying features of the problem. We should note that there was at times some inter-cluster similarity. However, the silhouette scores implied that more clusters resulted in compromised compactness and/or separation. According to our definition of the optimal feature set, we could allow for some correlation in cases where the clusters encapsulated relevancy. The feature importance factors, which we present later, showed that all of the clusters possessed strong relevancy to the problem at hand, except chull_orientation (which had some relevancy and no redundancy, so we did not exclude it from the optimal feature set as per our definition). One last note to mention is that the confidence half width of the group was 0.03, which did not drastically change the group similarity within the population.

The similarity coefficients were very strong in the first cluster, as portrayed by the first row of Table 6. Furthermore, we could put forward the same assertion for the entire population, as the maximum half width confidence was around 0.01. Since the cluster was purely geometric, we could take sinuosity or emax as comprehensible expressions of the underlying features that the cluster signified.

The second cluster possessed a somewhat strong core with a rather weak layer, which consisted of total_curvature, *average_turn_rate*, and turning_segments_proportion, surrounding it. The second row of Table 6 presents the descriptive statistics of the core. The mean and standard deviation of the similarity links between the layer and the core were 0.49 and 0.1, respectively. The characteristics of the cluster within the population resembled that within the sample. As the average and maximum half width of the confidence interval for the core were both 0.01 and both values for the layer were 0.02, the cluster consisted of a mixture of geometric and kinematic descriptors, so tagging the cluster with a single attribute that it would ultimately signify was nottrivial.

The third cluster contained all of the distance geometries; therefore, the interpretation that we presented when discussing the geometric problem applied here as well, just to a lesser extent, as shown by the third row of Table 6, which demonstrates that the similarity bonds between the distance geometries for all classes were weaker. The maximum half width of the confidence interval was just 0.02, which testified to the similarity bonds between the distance geometries within the population remaining comparatively strong.

The third cluster also included chull_aspect_ratio, with an average and standard deviation of the similarity between the distance geometries of 0.44 and 0.05, respectively; hence, it emerged as a rather weak link within both the sample and the population, as well as having a half width of 0.02. Regarding the expression of the underlying features, we could take any signature of the distance geometry (perhaps distance_1_1 because of its superior interpretability) as the representative of the underlying feature that the cluster signified due to the heavy presence of distance geometries and the rather weak similarity bond of chull_aspect_ratio.

The fourth cluster was somewhat strong, as shown by the fourth row of Table 6. The maximum half width of the confidence interval was 0.02, which rendered the cluster quite consistent within the population as well. We could see that the geometric and kinematic descriptors were quite entangled within the cluster, which implied that it was not possible to easily characterize the underlying features.

The fifth row of Table 6 presents the descriptive statistics of the fifth cluster. Although high_speed_proportion had a very low similarity bond to medium_speed_proportion (0.28), the first quartile showed that high_speed_proportion was similar to the rest of the cluster, for the most part. So, we could regard this cluster as reasonably cohesive as well. This assertion could also be made for the entire population, considering that the maximum half width of the confidence interval for the cluster was 0.02.

**Figure 12:**
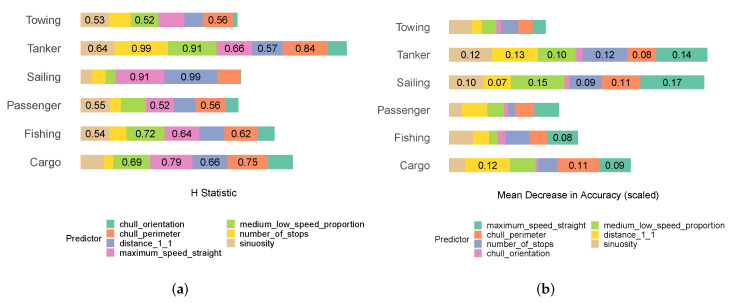
(**a**) The H test results for the predictors across all classes; (**b**) the mean decrease in accuracy (MDA) values for the predictors across all classes.

The permutation importance factors for maximum_speed_straight, distance_1_1, sinuosity, chull_perimeter, number_of_stops, medium_low_speed_proportion, and chull_orientation were 0.29, 0.28, 0.23, 0.22, 0.2, 0.19, and 0.06, respectively. As argued in Section 2.6, we employed the Kruskal–Wallis H test to explore the relationships between the descriptors in our hybrid model. To do so, we first reflected on Figure 12a in conjunction with Figure 12b, which illustrate the H test results and scaled mean decrease in accuracy (MDA) values for all classes, respectively. We scaled the MDA values with respect to the total number of instances of each class to provide more interpretable numbers. Figure 12a indicates that only chull_orientation did not rely on the values of other predictors. On the other side of the spectrum, the predictive power of maximum_speed_straight and chull_perimeter very much relied on the values of the other predictors.

**Figure 13:**
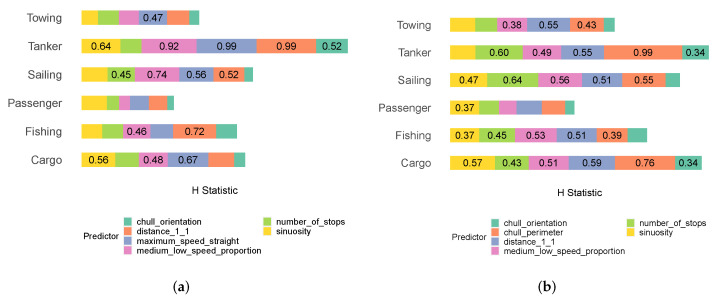
(**a**) The two-way H test results for chull_perimeter across all classes; (**b**) the two-way H test results for maximum_speed_straight across all classes.

We then investigated the two-way H tests of some example predictors to determine whether geometric and kinematic predictors could cooperate with each another to generate the response value. Figure 13a,b illustrate the two-way H test results of chull_perimeter and maximum_speed_straight, which were integral geometric and kinematic predictors. They were integral in the sense that they were both important and reliant on the values of the other predictors. The H test results indicated that chull_perimeter relied more on kinematic descriptors and maximum_speed_straight relied more on geometric descriptors. In fact, in the case of towing ships and sailing ships, the two descriptors that chull_perimeter relied on the most were kinematic. In the case of cargo ships, tankers, and fishing ships, the most relied upon descriptor was kinematic. In other cases too, kinematic descriptors were still quite integral to chull_perimeter. In the case of fishing ships after distance_1_1, the three next relied upon values were geometric: (maximum_speed_straight, number_of_stops, and medium_low_speed_proportion). With regard to maximum_speed_straight, the three most relied upon values in the case of passenger ships were geometric (sinuosity, distance_1_1, and chull_perimeter). Likewise, the two most relied upon descriptors in the case of towing ships, passenger ships, and cargo ships were geometric. In the case of sailing ships and fishing ships, maximum_speed_straight relied on a combination of geometric and kinematic predictors. The evidence attested to the fact that the geometric and kinematic descriptors were heavily reliant upon each another in carrying out predictions when all six classes were included.

### 3.3. Universal Similarity among Geometric and Kinematic Descriptors (RQ3)

In this subsection, we present the results for our third research objective surrounding universal similarity, in which we verified whether there were similar geometric and kinematic descriptors regardless of which of the six classes were included in a classification problem.

**Figure 14:**
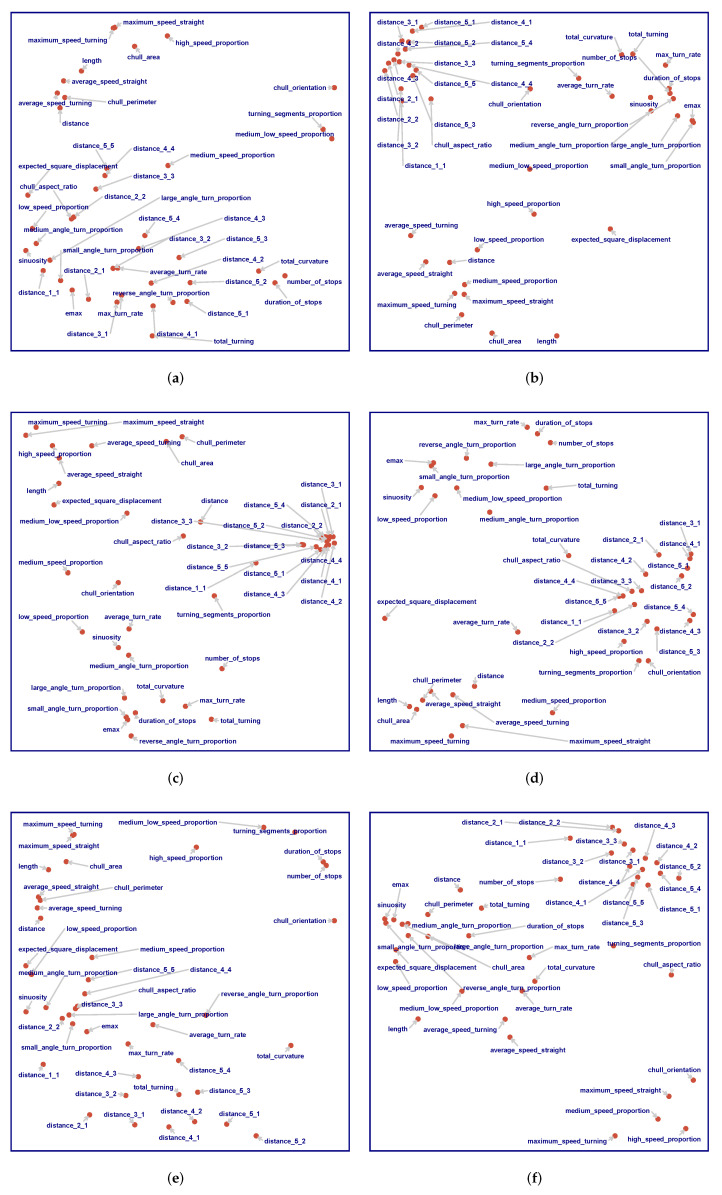
The 2D multidimensional scaling of the descriptors using data points that belonged to each: (**a**) cargo ships; (**b**) fishing ships; (**c**) passenger ships; (**d**) sailing ships; (**e**) tankers; (**f**) towing ships.

Although the correlations between the descriptors changed with what classes were included, we found that universal similarity did exist among the descriptors. For example, as the perimeter of the convex hull of the trajectory grew, so did its area (albeit not necessarily linearly). As outlined in Section 2, we then resorted to the 2D multidimensional scaling (MDS) of the descriptors using data points that belonged to each class to form a conjecture regarding universal similarity. This produced six maps, as illustrated by Figure 14.

**Figure 15:**
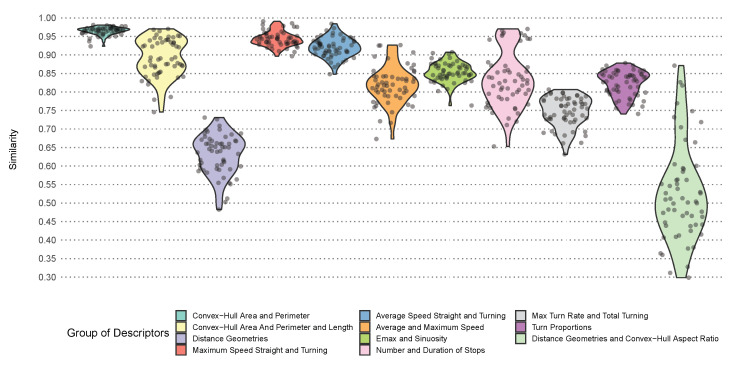
The verification of the conjecture regarding universal similarity. Each dot shows the average strength of the similarity bonds between several groups of predictors for each of the 57 problems.

Next, with reference to Figure 14, we formed a conjecture and verified it, as shown in Figure 15, in which the average strength of the similarity bonds between the conjectured group of predictors for each of the 57 problems are depicted. Even without observing Figure 14, we could safely assume that chull_area and chull_perimeter monotonically and universally increased and decreased together. On the other hand, length came very close to chull_area and chull_perimeter in the case of sailing ships. In the worst-case scenario for passenger and towing ships, the length was still close to the two descriptors, which suggested that a healthy universal bond of similarity could exist among the three. Figure 15 shows that the conjecture held as the similarity bond was strong as 0.8 to 0.95 (with a maximum half width of the confidence interval of 0.03). Without length in the mix, the average similarity stayed above 0.95.

The plots in Figure 14 suggested that maximum_speed_straight and maximum_speed_ turning could be universally similar. On the same note, the similarity between average _speed_straight and average_speed_turning, though weaker in some cases (such as passenger ships), might hold universally. This hypothesis was correct. The similarity bonds for both pairs, according to Figure 15, was between 0.90 and 0.95 in most cases (with a maximum confidence interval half width of 0.01 and 0.02, respectively). In fact, except towing ships, all four descriptors appeared within the vicinity of each another for all of the ship types. Figure 15 shows the strength of the similarity bonds in question, which fell between 0.75 and 0.90 for the most part (with a maximum confidence interval half width of 0.03).

The problems that we studied in the previous subsections insinuated that the similarity bonds between the distance geometries might be universal. However, Figure 14 somewhat undermines this theory, as the distance geometries for cargo ships and tankers were quite scattered. Both observations added up well as the similarity bonds only existed weakly in the majority cases (between 0.55 and 0.70, with a rather significant maximum confidence interval half width of 0.07).

Figure 14 suggested that emax and sinuosity might be universally similar. In fact, this was the case in Figure 15. For the most part, the strength of the similarity bonds were between 0.80 to 0.90, with a maximum confidence interval half width of 0.02. In the case of max_turn_rate and total_turning, there was a decent universal similarity with a strength of between 0.70 and 0.80, with a maximum confidence interval half width of 0.02, as shown in Figure 15. Interestingly, number_of_stops and duration_of_stops also emerged as extremely close for several ship types, as shown in Figure 14. Figure 15 showed that the conjecture could be a bit overestimated. That said, the bond was still as strong as 0.70 for most cases (with a maximum confidence interval half width of 0.02). In the geometric problem that we studied, the proportions of the different turn angles appeared within the same cluster. Figure 14 revealed that similarity bonds existed for all ship types (except for tankers, for which reverse_angle_turn_proportion was separate from the rest of the angle proportions). Figure 15 indicated that a universal similarity held for these descriptors as well. The average similarity bond for this group was at least 0.75 for the most part, with the significant maximum confidence interval half width of 0.05.

Some other descriptors that showed up within each others’ proximity in Figure 14 failed to be universally similar. For example, distance_1_1 (which could more or less be taken as the representative for the distance geometries) and chull_aspect_ratio were not universally similar, as shown in Figure 15.

Our investigation into the third question confirmed our main conjecture, i.e., there there were universally similar geometric and kinematic descriptors. As each group of similar descriptors, except one, was purely geometric or kinematic (the mixed group consisted of max_turn_rate and total_turning), these descriptors could be employed as alternatives when either geometric or kinematic descriptors were not available (for the reasons that we mentioned in Section 1).

## 4. Discussion and Conclusions

In this article, we provided a thorough analysis of the geometric and kinematic descriptors for the classification of ship trajectories into the following classes: cargo ships, tankers, towing ships, fishing ships, passenger ships, and sailing ships. In this section, we summarize our results and their implications within a broader context.

We investigated how much predictive accuracy was lost when either kinematic or geometric descriptors were not available. The results revealed that depending on the ship classes that were included in the classification problem, the predictive performance of models that were based on geometric descriptors and kinematic descriptors could vary significantly in relation to that of the benchmark models.

Our investigation showed that neither geometric nor kinematic descriptors outperformed the other in any of the possible classification problems in terms of predictive performance. In some cases, however, the geometric or kinematic models exhibited predictive performances that were close to those of the benchmark models. This implied that in situations when geometric or kinematic predictors were not available, acceptable models could still be available, depending on the classification problem at hand and the error tolerance. In several cases, the predictive performance suffered severely in the absence of either geometric or kinematic descriptors. This showed that in certain cases, geometric and kinematic descriptors could not replicate the results of the other type of descriptors.

Next, we examined the extent to which the geometric and kinematic descriptor could characterize differences between the movements of the different ship classes. This geometric or kinematic characterization (when simple enough) could assist marine data analysts in their knowledge acquisition process. In particular, we demonstrated that the geometric and kinematic descriptors could provide accurate and simple movement characterization at two levels, depending on the ship classes in question. The simplest characterization was possible when the predictive performance of either the geometric or kinematic descriptors approached that of the benchmark model. In which case, the differences in the characteristics could simply be ascribed to either the shape of the trajectory or the motion of the ship classes in question. At the second level, the optimal feature sets of the classification problems at hand could be identified. Through this, we demonstrated that it was possible to enhance the comprehension of the differences between the characteristics using specific geometric and kinematic properties. Nonetheless, the predictive performance of the geometric and kinematic descriptors in comparison to that of the benchmark models indicated that the characteristics of some problems did not manifest as solely geometric or kinematic. We further ascertained that in such cases, the geometric and kinematic descriptors could rely heavily on each another.

Lastly, we verified that a geometric–kinematic taxonomy could serve as a first step toward a basis for feature selection for ship classification. Feature selection in general can entail repetitive pick and choose processes between similar descriptors to increase the relevancy (to the classification) of the problem at hand and decrease the correlations among the predictors. A similarity-based taxonomy (possibly at several levels) could help practitioners to perform this procedure efficiently and effectively since it could suggest alternatives to certain descriptors or provide a group of descriptors to choose from that represent an underlying characteristic. In this light, our investigation suggested that a geometric–kinematic split might constitute the top level in such a taxonomy. The reason behind this inference was that several similar groups of descriptors *crystallized* within each class in each of the classification problems under study.

The multifaceted nature of the present study calls for research extensions in different directions. On a very general note, more descriptors could be included or the framework that was presented in this article could be applied to other ship classes (or even to classes of moving objects in other fields). Continuing with the general themes that were underlying the whole study, the choice of similarity measurements merits more investigation as the variety of perspectives that are offered by the many similarity measurements that are currently available could lead to further interesting findings [53]. Furthermore, with the prevalence of different trajectory compression approaches [54], it would be pertinent to investigate the extent to which the results of the present study hold for compressed trajectories, in which geometric and kinematic properties may be obscured.

There is also room for more extensions or improvements that are specific to each of the three research objectives. With regard to the predictive performance of the geometric and kinematic descriptors, it would be interesting to find out which classes have significant impacts on the performance of the geometric or kinematic descriptors. The techniques that were discussed in [55] stand out as potentially useful. Turning our attention to movement characterization, a deeper taxonomy with more levels could provide more nuanced characterizations. The existing literature on the more refined families of descriptors [2,3,6,12,20,21,30,56] renders multilevel taxonomy plausible. Lastly, in terms of the groups of universally similar descriptors, we only pointed to the compliance of universal similarity within a geometric–kinematic taxonomy. In this regard, further work could uncover all of the groups of universally similar descriptors with a reasonable degree of accuracy. The outcomes could equip practitioners with a global scheme for feature selection. The number and size of the hybrid groups could indicate how much correlation exists between the geometric and kinematic families. Mindful of that, practitioners could then pick and choose representatives from each group while maximizing the relevancy and minimizing the correlations.

## Figures and Tables

**Table 1:** A breakdown of the number of AIS messages and the number of trajectories according to class.

Ship Type	Number of AIS Messages	Number of Trajectories
Cargo	5,778,529	933
Tanker	3,313,088	731
Towing	14,460,834	1010
Fishing	14,385,974	1025
Passenger	3,935,171	753
Sailing	1,343,775	1027
Total	43,217,371	5479

**Table 2:** The list of geometric descriptors that were used in this study.

Descriptor	Identifier	Comment
Sinuosity	sinuosity	$1.18\sigma /\sqrt{q}$, where σ is the standard deviation of trajectory turning angles at each step and *q* is the mean step length
Distance geometries	distance_i_j for 1≤i≤5 and 1≤j≤i	A group of 1 + 2 + 3 + 4 + 5 = 15 descriptors that measure tortuosity as the effective distance (the ratio of the distance between the start and end points of a segment to the length of the segment); each term of the summation, which we call a signature, measures the tortuosity of the trajectory in progressively finer frequencies and then, the signatures together define the shape of a trajectory; therefore, the first signature consists of one descriptor and is the effective length of the entire trajectory, the second signature consists of two descriptors (the first being the effective length of the first segment and the second being the effective distance of the second segment), etc.; although the authors of [9] suggested the use of four signatures to capture enough variations, we opted for five to capture even more nuanced shapes
Distance	distance	Distance between the start and end points of trajectory
Maximum expected displacement of trajectory	emax	A dimensionless and scale-independent measure of trajectory straightness, as proposed by [38]; values closer to 0 indicate higher degrees of tortuosity, while larger values (approaching infinity) indicate lower degrees of tortuosity
Expected displacement of trajectory	expected_square_displacement	Values closer to 0 indicate a lower density of turning angles, while larger values (approaching infinity) indicate a higher density of turning angles [39]
Length of trajectory	length	The cumulative distance traveled along trajectory
Sum of absolute values of trajectory angles	total_curvature	Values closer to 0 indicate small course variations, while larger values (approaching infinity) indicate larger course variations
Sum of trajectory angles	total_turning	The sum of trajectory angles at each step
Proportion of small angles to total number of angles	small_angle_turn_proportion	Small angles constitute angles between $0^{\circ }$ and $45^{\circ }$
Proportion of medium angles to total number of angles	medium_angle_turn_proportion	Medium angles constitute angles between $45^{\circ }$ and $90^{\circ }$
Proportion of large angles to total number of angles	large_angle_turn_proportion	Large angles constitute angles between $90^{\circ }$ and $135^{\circ }$
Proportion of reverse angles to total number of angles	reverse_angle_turn_proportion	Reverse angles constitute angles between $155^{\circ }$ and $180^{\circ }$
Perimeter of convex hull of trajectory	chull_perimeter	Larger values imply longer trajectories; the convex hull of a trajectory is the smallest convex polygon within the trajectory
Area of convex hull of trajectory	chull_area	Larger values imply that the trajectory deviates more from the shortest path between the start point and end point of the trajectory; the convex hull of a trajectory is the smallest convex polygon within the trajectory
Ratio of shortest to longest axis of convex hull of trajectory	chull_aspect_ratio	The distance between the centroid of the convex hull and the nearest point on the convex hull and the longest axis is the distance between the centroid of the convex hull and the farthest vertex of the convex hull; smaller values imply more stretched out convex hulls
Orientation of convex hull of trajectory (with reference to hull’s longest axis)	chull_orientation	The longest axis is the distance between the centroid of the convex hull and the farthest vertex of the convex hull; we regarded the supplementary angles (adding up to $180^{\circ }$) as identical, so the orientation of the convex hull could be represented by a value between $0^{\circ }$ and $180^{\circ }$

**Table 3:** The list of kinematic descriptors that were used in this study.

Descriptor	Identifier	Comment
Average speed of turning	average_speed_turning	Average speed of the ship when it is turning
Maximum speed of turning	max_speed_turning	Maximum speed of the ship when it is turning
Average speed of straight sailing	average_speed_straight	Average speed of the ship when it is sailing straight
Maximum speed of straight sailing	max_speed_straight	Maximum speed of the ship when it is sailing straight
Maximum rate of turn	max_turn_rate	Rate as degrees per minute
Average rate of turn	average_turn_rate	Rate as degrees per minute
Proportion of trajectory in which the ship is turning with respect to entire trajectory	turning_segments_proportion	A value between 0 and 1 that indicates the ratio of accumulative duration of segments in which the ship is turning to entire duration of trajectory
Proportion of trajectory in which the ship is moving at up to 4 knots with respect to entire trajectory	low_speed_proportion	Based on [14], a value between 0 and 1 that indicates the ratio of accumulative duration of segments in which the ship is moving slower than 4 knots to entire duration of trajectory
Proportion of trajectory in which the ship is moving at 4 to 10 knots with respect to entire trajectory	medium_low_speed_proportion	Based on [14], a value between 0 and 1 that indicates the ratio of accumulative duration of segments in which the ship is moving between 4 to 10 knots to entire duration of trajectory
Proportion of trajectory in which the ship is moving at 10 to 18 knots with respect to entire trajectory	medium_speed_proportion	Based on [14], a value between 0 and 1 that indicates the ratio of accumulative duration of segments in which the ship is moving between 10 to 18 knots to entire duration of trajectory
Proportion of trajectory in which the ship is moving at more than 18 knots	high_speed_proportion	Based on [14], a value between 0 and 1 that indicates the ratio of accumulative duration of the segments in which the ship is moving faster than 18 knots to entire duration of trajectory
Number of anchored off segments	no_of_stops	An integer greater than or equal to 0 that counts the number of trajectory segments that are classed as anchored off; this descriptor can appear to be geometric at the first sight, but in the case of free-floating vessels, kinematic parameters determine whether the vessel is anchored off; in accordance with [14], in this study, when the location of the ship did not change for a certain period and the speed of the ship did not surpass a certain threshold, we then regarded the status of the ship as anchored off
Total time of anchored off segments	duration_of_stops	A value that is greater than or equal to 0, which is the sum of all duration values of each trajectory segment that are classed as anchored off; following the same argument as that presented for the previous descriptor, this descriptor is essentially kinematic in nature

**Table 4:** The descriptive statistics of the geometric descriptor-based, kinematic descriptor-based, and benchmark models, according to the classification problems.

Row No.	Min.	1st Qu.	Median	Mean	3rd Qu.	Max.	Description
1	0.0086	0.0387	0.0493	0.0512	0.0667	0.0945	The OOB errors produced by Geometric models minus those produced by the benchmark models.
2	0.0015	0.0309	0.0456	0.0452	0.0630	0.0760	The OOB errors produced by Kinematic models minus those produced by the benchmark models.
3	0.0214	0.0418	0.0545	0.0558	0.0693	0.0945	The OOB errors produced by the best alternative model (either Kinematic or Geometric) minus those produced by the benchmark models.
4	−0.0272	−0.0055	0.0069	0.0060	0.0184	0.0395	The OOB errors produced by the Geometric models minus those produced by the Kinematic models.

**Table 5:** The descriptive statistics of the similarity between the distance geometries of the cargo ships vs. passenger ships problem.

Min.	1st Qu.	Median	Mean	3rd Qu.	Max.
0.5700	0.6736	0.7231	0.7308	0.7636	0.9443

**Table 6:** The descriptive statistics of the similarity bonds within the clusters of the hybrid problem.

Row No.	Min.	1st Qu.	Median	Mean	3rd Qu.	Max.	Description
1	0.7764	0.8159	0.8837	0.8763	0.9325	0.9754	Similarities pertaining to the first cluster of the hybrid problem.
2	0.6791	0.6799	0.7036	0.7286	0.7758	0.8296	Similarities pertaining to the second cluster of the hybrid problem.
3	0.4843	0.5600	0.6201	0.6511	0.7270	0.9556	Similarities pertaining to the third cluster of the hybrid problem.
4	0.4842	0.6628	0.6907	0.7221	0.8246	0.9678	Similarities pertaining to the fourth cluster of the hybrid problem.
5	0.2795	0.6116	0.6800	0.6560	0.7422	0.9425	Similarities pertaining to the fifth cluster of the hybrid problem.

## Data Availability

The data used in this study is available for public use at https://marinecadastre.gov/ais/ (accessed on 13 June 2022).
